# Impact of mood disorders and personality disorders on suicide intent among self-harm patients

**DOI:** 10.12669/pjms.39.2.6140

**Published:** 2023

**Authors:** Naila Yaqoob, Sadaf Ahsan

**Affiliations:** 1 Major Dr. Naila Yaqoob, Clinical Psychologist, CMH Multan, Pakistan; 2 Dr. Sadaf Ahsan Associate Professor, Foundation University, Islamabad, Pakistan

**Keywords:** Mood Disorder, Personality Disorder, Suicide Intent, Self-harm Patients

## Abstract

**Objectives:**

The present research examined the impact of mood disorder and personality disorders on suicide intent among self-harm patients using cross sectional research design.

**Methods:**

A clinical sample of 220 self-harm inpatients with age of 18 to 35 years was collected through purposive sampling technique from different mental health departments of Rawalpindi, Jhelum and Multan hospitals. Study was conducted from June 2019 till November, 2021. Data was compiled in three steps. In step-I, rapport was established with patients and identification of intent was carried out by administering Beck Suicide Intent Scale. In step-II, mental illness was identified by administering Mood Disorder Questionnaire, Personality Diagnostic Questionnaire and subscale of Depression from Depression Anxiety and Stress Scale. In step-III, in order to confirm his / her responses and diagnosis, a detailed semi-structured clinical interview was carried out with each participant who responded positively to Personality Diagnostic Questionnaire.

**Result:**

Results revealed that bipolar spectrum disorder, depression and antisocial personality disorder were significantly positive predictors of suicide intent among self-harm patients. On the other hand, narcissistic personality disorder and borderline personality disorder were found to be significantly negative predictors of suicide intent among self-harm patients.

**Conclusion:**

Results of current research highlighted those predisposing factors that discriminate self-harm from attempted suicide. The study has important implications for identification of intent of self-harm patients, which could help in devising a proper treatment plan for the management of such challenging patients.

## INTRODUCTION

Self-harm is one of the most important concerns of public health across the world, as it is more prevalent common than completed suicide.^1^ The number of people who engage in self-harming behavior are 20 % greater in numbers than those who commit suicide. Unfortunately, the incidences of self-harm are increasing dramatically across the world and grasping attention from policy makers, researchers and health care professionals.^2^ The term self-harm refers to diversity of behaviors in which a person deliberately imposes harm to his or her own body; these behaviors are not socially acknowledged or allowed and regardless of underlying intent.^1^ Suicide, in current time, is the fourth primary cause of all deaths and occur mostly during young adulthood among U.S. children and teenagers aged between 10 to14 years, third midst between 15 to 24 years, and second amongst between 25 to 34 years. Presently, it is reported that annually around 800,000 people die from suicide, demonstrating a yearly international suicide ratio of 11.4 per 100,000. Suicide trends are more amongst males.^3^

In Pakistan like other developing countries there is an immense need to examine self-harm and associated features among adults. According to a research finding, there are about 100,000 acts of self-harm in Pakistan alone.^4^ It is thus imperative to determine the basic characteristics that give rise to self-harm and suicidal behaviors. Self-harm behavior occurs in nearly about 20% psychiatric adult patients and 40 to 80% adolescents and young adult psychiatric patients. Virtually 25% of youth, those who practice self-harm behaviors revealed that they had suicidal intent.^5^ Epidemiological studies revealed that among self-harm’ people around 90% individual suffered psychiatric ailment at the time of suicide attempt. Self-harm acts, suicidal ideation, suicide attempts and psychiatric ailments are predictors of future suicide.^6^ It is remarkably difficult to manage self-harm in clinical practice because of its nature of intent or motive behind that act, suicidal or non-suicidal; thus, inscribing it as a challenging area in clinical practice.^7^

DSM-5 properly identifies the sturdy link among mood disorders, self-harm and suicide risk and contains a suicide risk factor among mood disorders like bipolar and depressive disorder.^8^ Suicidal studies show that 60 percent of suicides are due to mood disorders; grief and sadness normally occur proximately prior and succeeding self-harm. Depression is more prevailing mental illness and is a serious ailment and if not properly handled, may end up with suicide.^9^ Depression is found in 45 to 70 percent of suicides cases and there is a high risk for complete suicide during its first episode. Comorbid depression with personality disorders surge the risk of suicide.^10^ According to DSM-V, personality disorders have been described as enduring pattern of maladaptive personality characteristics that lead to significant distress and functional impairment. Cluster B (comprising histrionic, narcissistic, borderline and antisocial personality disorders) are dramatic, emotional or erratic personality disorders. The cluster B of personality disorders in particular, was the focus for the present study. Self-harm is frequently found among patients identified with borderline personality disorder as a conveying indicator of inner confusion and is the common personality disorder in clinical settings that disturbs approximately 1–2% of the over-all population. Self-harm with suicidal intent had augmented rates of paranoid, dependent, antisocial, avoidant or histrionic personality disorders.^11^

Prevalence of mental disabilities in Pakistan has increased up to 34%. Pakistan, India and Sri Lanka are those three emerging Asian countries that account for world’s more than 100,000 (10%) deaths due to self-harm. In Pakistan, the incidence of mental disorders, self-harming behaviors, stressful happenings were commonly reported risk factors for suicidal behavior across case studies. Suicidal behavior and self-harm is an under-researched focus in Pakistan. The research work revealed that 1.12 out of 100,000 deaths were due to self-harm from 1998 to 2001.^12^ The number of people who do self-harm is far greater than suicide as there are about 100,000 acts of self-harm in Pakistan alone.^13^ Considerable support has been provided by literature that there is a need to study mental illness among psychiatric clinical self-harm population in Pakistan. The literature emphasized the need of precise identification of mood and personality disorder and suicide intent among self-harm patients. The current research contributes towards existing literature on self-harm while augmenting understanding of the issue by supporting the effectiveness of latest classification adopted by the DSM-5 i.e., self-harming behaviors are classified based on underlying intent. Thus, this study objective was to examine, the impact of mood disorders on suicide intent in patients of self-harm and further goals to examine the role of personality disorders with respect to suicide intent in self-harm patients.

## METHODS

A purposive sample of 225 Self-Harm patients was gathered from the emergency room of Mental Health Departments of Rawalpindi, Jhelum and Multan, hospitals. Study was conducted from June 2019 till November, 2021. The participants were divided into two different groups. Participants were placed into groups like those who scored above cut off i.e., 15 on Beck Suicide Intent Scale (BSIS) were placed in self-harm with suicide intent group. Participants were placed in the self-harm without suicide intent group if they showed no intent to die on BSIS. Sample comprised age range between 18 - 35 years (M= 26.44, SD=5.95), whether married or unmarried, having at least matric qualification (mean education was intermediate).

### Inclusion/ Exclusion Criteria:

Patients having at least one-year involvement in self-harm act were included. Self-harm in form of tattoo and/or culturally sanctioned behaviors was excluded. Participants having current active risk of self-harm or violence, having intellectual disability or psychotic disorders or with less than matriculation qualification were excluded. Participants other than depression, bipolar disorder and personality disorders were also excluded. Three participants, meeting the criteria of drug abuse disorders, two participants in psychotic phase of mood disorder were, thus, not included. Consequently, 220 participants were included in the research.

The main study was carried out on larger sample (N=220) to find out the impact of personality and psychopathology on suicide intent and underlying roles of social support, parental bonding and emotions with respect to suicide intent among self-harm patients. A sample of 220 Self-Harm patients (*n = 126 Females, n = 94 Males*) was collected from the emergency room and mental health departments of different hospitals. Data was collected in four steps. In step one, rapport was established and identification of intent was carried out by administering Urdu translated Beck Suicide Intent Scale. In step two, mood disorder was examined in terms of Bipolar Spectrum Disorder (which involves bipolar-I, bipolar-II and bipolar NOS) and depression by administering Urdu translated Mood Disorder Questionnaire and Depression subscale of Depression, Anxiety and Stress Scale. In step three, personality disorders were screened out by administering Personality Diagnostic Questionnaire, followed by a detailed semi structured clinical interview was carried out with each participant, responding positively to Personality Diagnostic Questionnaire and Mood Disorder Questionnaire, to confirm his/her responses and diagnosis. Each sitting required 30 to 45 minutes prolonged session per person to collect his data. First, ethical approval was obtained from Institutional Review Board (FURC/Psy/IRB/26 dated 11^th^ July, 2018). Moreover, permission from head of the departments and written consent from the participants were taken. APA guidelines were strictly followed while collecting data from patients. Their confidentiality was kept in mind and they were also offered counselling sessions after the completion of data collection, as many patients needed counselling sessions. Thus, therapeutic counselling sessions were conducted with patients and follow-up session were also carried out in which they gave positive feedback and showed improvement.

## RESULTS

Statistical Package for Social Sciences (IBM-SPSS Version 23) was used to analyze data of main study by using descriptive statistics. Descriptive analysis and item total correlations were computed to check internal consistency of the scales (N=220). To find relationship between variables, bivariate correlation analysis was used. Regression analysis was used to infer causal relationships between the variables.

The mean, standard deviation, range, skewness, kurtosis and Cronbach alpha reliability of each scale and subscales is shown in Table-I. Cronbach alpha reliabilities of Beck Suicide Intent Scale (X̄ =13.65*, SD* = 6.53, *α* = .85) and Mood Disorder Questionnaire (X̄ =6.79*, SD* = 4.33, *α* = .84) are in acceptable range. Overall, results indicate all scales have acceptable reliabilities coefficients. Standard deviation of each scale was observed as neither too high nor too small and was a reasonably spread around mean which shows that the data is normally distributed. Findings of skewness showed that the data was normally distributed.

**Table-I:** Descriptive Statistics for all Scales and their Subscales (N=220)

Scales	k	α	M	S.D	Range		Skew	Kurt

					Actual	Potential		
BSIS	15	.85	13.65	6.53	0-25	0-30	.07	-.66
MDQ	15	.84	6.79	4.33	1-15	0-17	.03	-.07
***Depression Anxiety and Stress Scale (DASS)***								
Depression	14	.91	25.00	9.28	0-21	0-21	-.65	-.37
Anxiety	14	.89	21.82	8.33	1-20	0-21	-.07	-.61
***Personality Diagnostic Questionnaire (PDQ)***								
HPD	08	.69	4.37	1.77	1-5	0-8	.15	-.78
NPD	09	.76	4.53	2.59	0-7	0-9	-.99	-.88
BPD	14	.77	6.92	3.01	1-9	0-14	-.12	-.75
APD	07	.69	4.05	1.88	1-6	0-14	-.17	-.99

***Note:*** M = Mean, S.D = Standard deviation, α = Cronbach alpha reliability, BSIS= Beck Suicide Intent Scale, MDQ= Mood Disorder Questionnaire, HPD= Histrionic Personality Disorder, NPD= Narcissistic Personality Disorder, BPD= Borderline Personality Disorder, APD=Antisocial Personality Disorder.

The correlation coefficient between study variables is shown in Table-II. Bipolar spectrum disorder, depression and antisocial personality have significantly positive correlation with suicide intent thus depicting that with the increase in bipolar spectrum disorder, depression and antisocial personality disorder, suicide intent also increases among self-harm patients. Narcissistic personality disorder and borderline personality disorder have significant negative correlation with suicide intent among self-harm patients. Depicting narcissistic personality disorder and borderline personality disorder decreases the risk of suicide intent among self-harm patients.

**Table-II:** Bivariate Correlation among Study Variables (N=220).

Variables	1	2	3	4	5	6	7
Histrionic PD	-	.30**	.20**	.57**	.16*	-.07	.04
Narcissistic PD		-	.39**	.28**	-.01	-.24**	-.35**
Borderline PD			-	.12	-.33**	-.50**	-.48**
Avoidant PD				-	.39**	.20**	.23**
Bipolar Spectrum Disorder					-	.39**	.43**
Depression						-	.71**
Suicide Intent							-

***Note.***

* p<.05;

** p<.01; ***p<.000, PD= Personality Disorder.

Bipolar spectrum disorder and depression significantly predicted suicide intent in positive direction, thus, revealing that depression and bipolar spectrum disorder increase suicidal risk among self-harm patients. Table-III.

**Table-III:** Suicide Intent predicted by Psychopathology (Depression, Anxiety and Bipolar Spectrum Disorder) in self-harm patients (N =220).

Variables	B	S.E	β	t	P	R^2^	R^2^_adj_
***Predictor***							
(Constant)	1.86	0.95		1.96		0.56	0.55
Bipolar Spectrum Disorder	0.28	0.07	0.19	3.77	0.00		
Depression	0.53	0.04	0.75	12.77	0.00		

***Note.*** *p<.05; **p<.01.

Narcissistic personality disorder and borderline personality disorder significantly predicted suicide intent in negative direction and accounted for 33% variance in suicide intent. Table-IV. It thus clarifies that narcissistic personality disorder and borderline personality disorder decreases risk of suicide among self-harm patients. Whereas, histrionic personality disorder and antisocial personality disorder predicted suicide intent in positive direction being high indicators of suicide intent.

**Table-IV:** Suicide intent predicted by Personality disorders (Histrionic personality disorder, Narcissistic personality disorder, Borderline personality disorder, Anti-social personality disorder) in self-harm patients (N =220).

Variables	B	S.E	Β	t	p	R^2^	R^2^_adj_
***Predictor***							
(Constant)	17.98	1.18		15.21	.18	0.38	0.37
Histrionic PD	0.05	0.25	0.01	.21	.84		
Narcissistic PD	-0.73	0.15	-0.29	-4.74	.00		
Borderline PD	-0.89	0.13	-0.41	-7.01	.00		
Avoidant PD	1.22	0.23	0.35	5.32	.00		

***Note.*** *p<.05; **p<.01, PD= Personality Disorder

## DISCUSSION

The manifestation of self-harm behaviors is on the rise around the globe. Understanding the phenomenon in which an individual deliberately chooses to harm his or her own life, in order to get escape from agonizing pain, or lose a desire to live, has perplexed researchers or clinicians for times. There is a big need of determining the basic characteristics that give rise to suicidal behaviors. The current results are aimed to determine the impact of mood disorders and personality disorders on suicide intent among self-harm patients.

Self-harming behaviors are commonly reported in health care system. A study conducted among people who had contact with mental health services, reported that nearly half had harmed themselves in the last six months.^6^ Likewise; patients with self-harm and admitted in mental health departments were target for current research. Results of present research revealed that out of 220 self-harm patients, ninety-three patients reported to had suicide intent at the time of self-harm behavior. Correlation analyses of current research also revealed that bipolar spectrum disorder, depression and antisocial personality had significant positive correlation with suicide intent among self-harm patients. It was thus, indicated that self-harm patients with bipolar spectrum disorder, depression and/or antisocial personality disorder had more suicide intent. Whereas, narcissistic personality disorder and borderline personality disorder, have significant negative correlation with suicide intent among self-harm patients thus depicting that narcissistic personality disorder and borderline personality disorder decrease the risk of suicide intent among self-harm patients. Another study found suicide intent among self-harm patients was very less among patients having personality disorders comparatively to patients having other mental disorders.^11^

A previous research found bipolar disorder, depression and psychiatric comorbid disorders were significantly related to suicide intent among self-harm.^14^ Studies have identified that those who self-harm without suicidal intent are more associated with borderline personality disorder and narcissistic personality disorder.^15^-^17^ Moreover, Ramleth and his colleagues in 2017 also found less suicide ideation among self-harm patients with borderline personality disorder.^18^ In addition, self-harm patients with personality disorders had very less suicide intent than patients with other mood disorder diagnoses.^19^

Results of current research are consistent with existing empirical evidence. In addition, regression analyses revealed that bipolar spectrum disorder, depression, histrionic personality disorder and antisocial personality disorder significantly predicted suicide intent in positive direction thus revealing that depression, bipolar spectrum disorder, histrionic personality disorder and antisocial personality disorder increased suicidal risk among self-harm patients. Results also revealed that narcissistic personality disorder and borderline personality disorder predicted suicide intent in negative direction thus indicating that narcissistic personality disorder and borderline personality disorder decrease risk of suicide among self-harm patients. Similarly, Ferrara, Terrinoni and Williams, conducted research on personality features and attitude toward death among self-harm inpatients and found 63.5% met criteria for borderline personality disorder and 46.1% revealed having depression.^20^

In study of Muehlenkamp and his colleagues in 2011 found co-morbid antisocial personality disorder or borderline personality disorder, however, is more highly associated with self-harm.^16^ Impulsive and antisocial traits may differentiate self-harm with suicide intent from without suicide intent. In males, the impulsivity and antisocial personalities of psychopathology are intensely related with self-harm regardless of the other psychopathy characteristic.^15^ Haw and colleagues found that 51% of the people who harm themselves had a personality disorder at the time of the attempt.^17^ conclusively, psychopathological personality traits are associated with self-harm behaviors. Self-injurers are more expected to be identified with a mood disorder, like major depressive disorder.^21^ Mood disorders are well-known established predictor in suicides. Among 229 suicides, a psychological analysis revealed 44 percent people had mood disorder; 31 percent were those who had mood disorder and personality disorder diagnoses and 50 percent were those who had mood disorder and general medical diagnoses.^22^ Findings of present research are consistent with previous studies.

### Limitations:

Firstly, this study relied on results of a cross section research design. Secondly, self-harm needs to be further explored in patients with complex mental health needs (for instance psychosis or having comorbid issues such as drug addicts). Future researchers therefore, might benefit from focusing on these variables and should be investigated in future research.

### Suggestions:

The current research examined the impact of mood disorders and personality disorders with suicide intent among self-harm patients. Data obtained from patients with self-harm reporting to the psychiatric departments is confidential, sensitive and a challenging task, as it required proper rapport building to break defenses to rule out psychopathology.

## CONCLUSION

Results of current research aligning with recent literature and a big support for the classification of self-harming behaviors based on underlying intent as proposed by DSM 5, 2013 among patients with self-harm, as it is more challenging than originally thought. Results of current research have established that mood disorders and personality disorders are important factors in predicting suicide intent among self-harm patients.

### Authors’ Contribution:

**NY:** Conceived, designed and did data collection, statistical analysis & writing of manuscript, is responsible for integrity of research.

**SA:** Did review and final approval of manuscript.
